# TIPE2 acts as a tumor suppressor and correlates with tumor microenvironment immunity in epithelial ovarian cancer

**DOI:** 10.18632/aging.204529

**Published:** 2023-02-17

**Authors:** Shuai Xu, Xiaolin Gao, Jianqing Qiu, Fanzhen Hong, Fufeng Gao, Xia Wang, Shiqian Zhang

**Affiliations:** 1 Department of Obstetrics and Gynecology, Qilu Hospital, Cheeloo College of Medicine, Shandong University, Jinan 250012, Shandong, China; 2 Department of Obstetrics and Gynecology, The Second Hospital, Cheeloo College of Medicine, Shandong University, Jinan 250033, Shandong, China; 3 Department of Gynecological Oncology, Shandong Cancer Hospital and Institute, Shandong First Medical University and Shandong Academy of Medical Sciences, Jinan 250117, Shandong, China; 4 Laboratory of Translational Gastroenterology, Department of Gastroenterology, Qilu Hospital, Cheeloo College of Medicine, Shandong University, Jinan 250033, Shandong, China

**Keywords:** epithelial ovarian cancer, TIPE2, progression, PI3K/Akt signaling pathway, tumor microenvironment

## Abstract

Background: Epithelial ovarian cancer (EOC) is one of the deadliest gynecologic cancers. The etiology of EOC has still not been elucidated thoroughly. Tumor necrosis factor-*α*-induced protein 8-like2 (TNFAIP8L2, TIPE2), an important regulator of inflammation and immune homeostasis, plays a critical role in the progression of various cancers. This study aims to investigate the role of TIPE2 in EOC.

Methods: Expression of TIPE2 protein and mRNA in EOC tissues and cell lines was examined using Western blot and quantitative real-time PCR (qRT-PCR). The functions of TIPE2 in EOC were investigated by cell proliferation assay, colony assay, transwell assay, and apoptosis analysis *in vitro*. To further investigate the regulatory mechanisms of TIPE2 in EOC, RNA-seq and western blot were performed. Finally, the CIBERSORT algorithm and databases including Tumor Immune Single-cell Hub (TISCH), Tumor Immune Estimation Resource (TIMER), Tumor-Immune System Interaction (TISIDB), and The Gene Expression Profiling Interactive Analysis (GEPIA) were used to elucidate its potential role in regulating tumor immune infiltration in the tumor microenvironment (TME).

Results: TIPE2 expression was shown to be considerably lower in both EOC samples and cell lines. Overexpression of TIPE2 suppressed EOC cell proliferation, colony formation, and motility *in vitro*. Mechanistically, TIPE2 suppressed EOC by blocking the PI3K/Akt signaling pathway, according to bioinformatics analysis and western blot in TIPE2 overexpression EOC cell lines, and the anti-oncogenic potentials of TIPE2 in EOC cells could be partially abrogated by the PI3K agonist, 740Y-P. Finally, TIPE2 expression was positively associated with various immune cells and possibly involved in the regulation of macrophage polarization in ovarian cancer.

Conclusions: We detail the regulatory mechanism of TIPE2 in EOC carcinogenesis, as well as how it correlates with immune infiltration, emphasizing its potential as a therapeutic target in ovarian cancer.

## INTRODUCTION

Ovarian cancer (OC) is the most lethal gynecological cancer among women, ranking as the 7th most common malignancy and the 2nd leading cause of death from gynecologic cancer worldwide [1]. Data from the GLOBOCCAN database, a global cancer observatory, indicated that OC caused 313,959 new cases and 207,252 deaths globally in 2020 [2]. Despite the higher incidence of cervical and endometrial cancer, OC is responsible for more deaths than any other malignancy of the female genitourinary system. A review of publications from the Scopus database, Medline, and Web of Science Core Collection indicated that ovarian cancer was responsible for 4.4% of all cancer-related mortality in women [3]. The prognosis of cancer hinges on prompt diagnosis and the availability of systemic treatment. According to a recent study, survival rates for most malignancies have increased since the mid-1970s, which may be credited primarily to cutting-edge research, improvements in screening methods, and better treatment regimens, including the application of targeted therapies [4]. However, the global OC incidence is anticipated to rise to 434,184 new cases and 293,039 fatalities by 2040 [5]. Over 90% of OC cases originate from the epithelium, which is known as epithelial ovarian cancer (EOC) [6]. EOC is typically asymptomatic in its early stage, and over 75% of EOCs cases are found at an advanced stage when the tumor metastasizes to the perineum, which is classified as stage IIIC and IV cancer by the International Federation of Gynecology and Obstetrics (FIGO) [7]. This substantially increases mortality since the 5-year survival rate drops when cancer metastasizes to the pelvic cavity. According to the American Congress of Obstetricians and Gynecologists (ACOG), most EOCs have a 5-year survival rate of 20% to 30% [8], with most deaths occurring within 2 years of diagnosis [9]. As a result, thorough research into the underlying mechanisms and molecular profiles of EOC is urgently needed to provide new targets and therapeutic approaches.

While the etiology and pathophysiology of OC are poorly understood, several investigations have proven a link between chronic inflammation and cancer [10–12]. Inflammation is essential for maintaining physiological homeostasis and body stabilization. It is also estimated to account for around 25% of cancer-causing factors [13]. An imbalance between pro- and anti-inflammatory mediators may cause self-tissue damage, leading to gene mutations and aberrant cellular proliferation [14]. Chronic inflammation causes inflammatory and epithelial cells to produce reactive oxygen/nitrogen species (ROS/RNS), which would promote DNA aberration, and mutagenesis, inactivate tumor suppressor genes such as p53 and promote cellular proliferation and oncogenesis [15]. Inflammation also enhances intra-organ malignant cell migration [16]. Several studies demonstrate that variables related to inflammation of ovarian epithelium, such as pelvic inflammatory diseases, endometriosis, and ovulation are all linked to EOC [17]. For many years, the treatment of malignant tumors has focused on tumor cell elimination. Recent studies suggest that strategies aimed at altering inflammation in the TME may offer innovative therapeutic strategies to improve patient outcomes. Anti-inflammatory molecules may be potential candidates since they can function by modifying the host microenvironment.

Studies noted that immune cells can secrete tumor necrosis factor-alpha (TNF-α) in response to inflammation. TNF-α binds to tumor necrosis factor receptor types 1 (TNFR1) and 2 (TNFR2), triggers the nuclear Factor-B (NF-κB) signaling pathway, and increases the production of tumor necrosis factor-α-induced protein 8 (TNFAIP8/ TIPE) family proteins [18]. The role of the TIPE family in TNF-α regulated biological processes such as cancer and immunology has recently captured a lot of attention. Members of the TIPE family include TNFAIP8, TNFAIP8-like 1 (TIPE1), TNFAIP8-like 2 (TIPE2), TNFAIP8-like 3 (TIPE3) [19], with TIPE2 being a recently found negative regulator of immunity expressed mostly in inflammatory and lymphoid tissues [20]. Further studies demonstrated a constitutive TIPE2 expression observed in macrophage, B, and T lymphocytes [21]. In addition, TIPE2 has been shown to be expressed in a variety of endocrine and germ cells in mice [22]. By negatively modulating T cell receptor (TCR) and Toll-like receptor (TLR) signaling, TIPE2 is a crucial regulator of immunological homeostasis in both cellular and innate immunity [23]. TIPE2 knockout in mice was found to induce leukocyte aggregation, splenomegaly, over-reactivity, multi-organ inflammation, inflammatory diseases, and increased serum inflammatory cytokine levels such as interleukin (IL)-1, IL-6, IL-12, and TNF-α [24], indicating its importance in the immune homeostasis maintenance.

In addition to modulating inflammation, TIPE2 also acts as an inhibitor of oncogene Ras and an important regulator in carcinogenesis and TME during oncogenic progression [25]. TIPE2 has been studied for its potential utility as a biomarker in various cancer types. Its expression was shown to be low in human hepatocellular carcinoma [26], glioma tissues [27], prostate cancer [28], gastric cancer [29], breast cancer [30, 31], and small cell lung cancer [32]. Mechanistically, TIPE2 could bind with RalGDS domains, thereby preventing Ras from creating an active complex and limiting the activation of Akt and Ral (downstream effectors), thus preserving the dynamic balance between cell survival and apoptosis. Overexpression of TIPE2 increased cell apoptosis and greatly reduced Ras-induced carcinogenesis, indicating its potential role as a neoplastic disease suppressor. TIPE2 has also been found to reduce epithelial-mesenchymal transition (EMT) and metastasis through suppressing Wnt/β-catenin and PI3K/Akt signaling pathway [28, 33]. TIPE2 also elicits its anti-tumor activity by activating T and NK cells while inhibiting FoxP3+ Treg cells in the TME [34, 35]. However, the function of TIPE2 in EOC and the relationship between TIPE2 and tumor immunity in EOC is largely unknown.

In this study, we analyzed the expression profile, biological function, and associated signaling pathways of TIPE2 in EOC. Furthermore, the relationship between TIPE2 and TME was investigated using sequencing datasets. The variable expression and distinct function of TIPE2 imply that it may be used as a diagnostic biomarker, prognostic indicator, and therapeutic target for EOC.

## RESULTS

### TIPE2 expression is significantly decreased in human EOC

**Figure 1:**
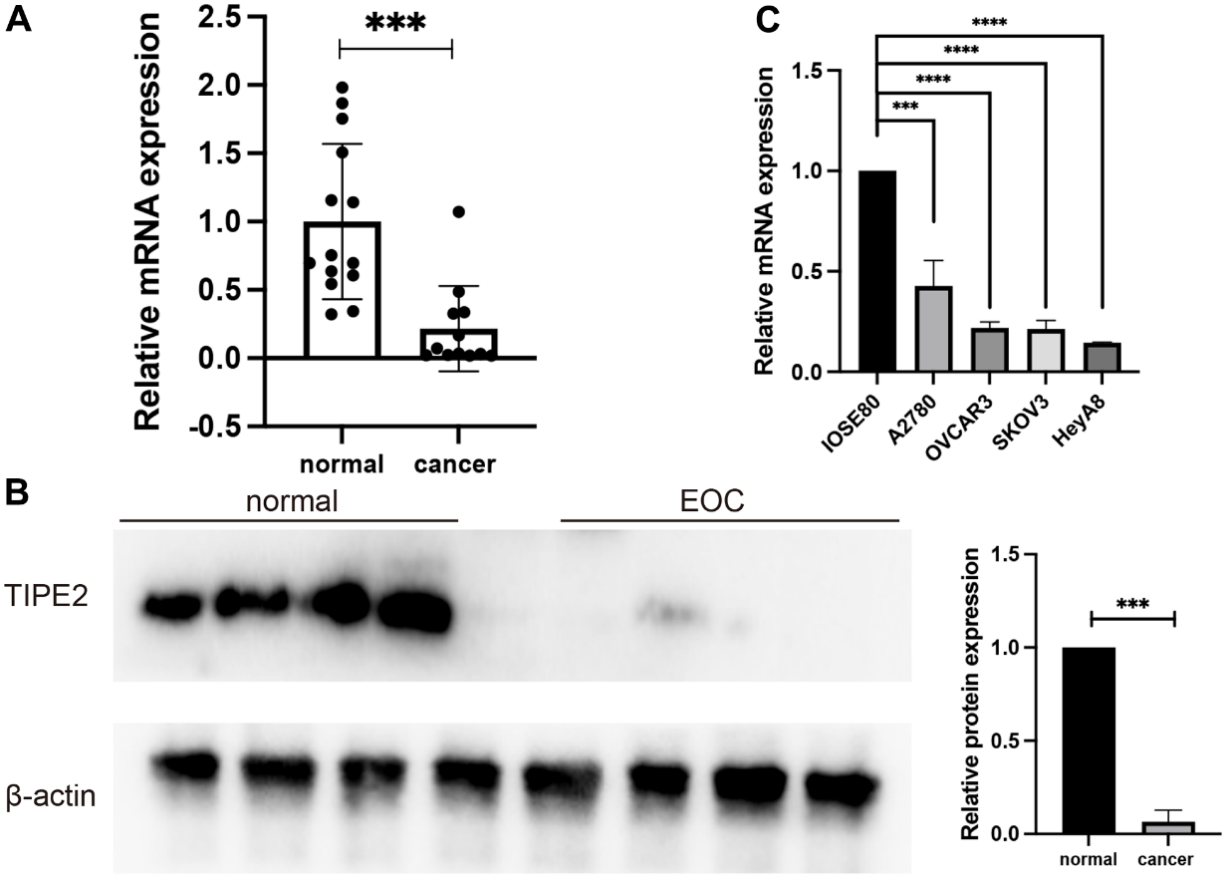
**TIPE2 expression was decreased in human EOC.** (**A**) qRT-PCR was used to measure TIPE2 mRNA expression in clinical samples from EOC (n=12) and non-cancer individuals (n=14). TIPE2 mRNA was shown to be considerably lower in EOC patients than in normal ovarian tissues. (**B**) Western blot examination of the relative expression of TIPE2 in EOC (n=4) and normal control samples (n=4). TIPE2 expression was normalized to β-actin. (**C**) qRT-PCR was conducted to assess TIPE2 RNA levels in IOSE80, a non-tumorigenic human-derived ovarian cell line, and 4 EOC cell lines, A2780, OVCAR3, SKOV3, HeyA8 (n=3). β-actin was used as an internal control. Data are presented as mean± SD. Statistical significance is denoted by the following symbols: ***p < 0.001, ****p < 0.0001.

The expression of TIPE2 in EOC has not yet been elucidated. To investigate the expression profile of TIPE2 in EOC, we initially collected 12 EOC tumor tissues and 14 normal ovarian tissues. The clinicopathological characteristics of the patients were listed in Table 1. The qRT-PCR and Western blot analysis indicated that TIPE2 is considerably underexpressed in the majority of EOC tissues as compared to normal ovarian tissues (Figure 1A, 1B). qRT-PCR was also used to assess TIPE2 expression in a panel of EOC cell lines, including A2780, OVCAR3, SKOV3, HeyA8, and IOSE80, a non-tumorigenic human ovarian cell line. In consistent with the expression profile in EOC tissues, TIPE2 presented a lower expression in EOC cell lines in comparison with IOSE80 cells at the mRNA level (Figure 1C). Taken together, our findings suggest that TIPE2 may serve as a predictive factor for tumor progression in EOC.

**Table 1:** Clinicopathological characteristics in 12 patients of EOC and 14 normal control patients.

**Variables**	**Patients of EOC** **(n=12)**	**Normal control patients** **(n=14)**
Age (years)		
< 60	8 (66.67%)	9 (64.29%)
≥ 60	4 (33.33%)	5 (35.71%)
FIGO stage		
I/II	7 (58.33%)	
III/IV	5 (41.67%)	
Grade		
1	3 (25%)	
2	4 (33.33%)	
3	5 (41.67%)	
Tumor type		
Serous	10 (83.33%)	
Mucinous	2 (16.67%)	
Lymph node metastasis		
No	8 (66.67%)	
Yes	4 (33.33%)	

### TIPE2 inhibits the proliferation and promotes apoptosis of EOC cells *in vitro*

**Figure 2:**
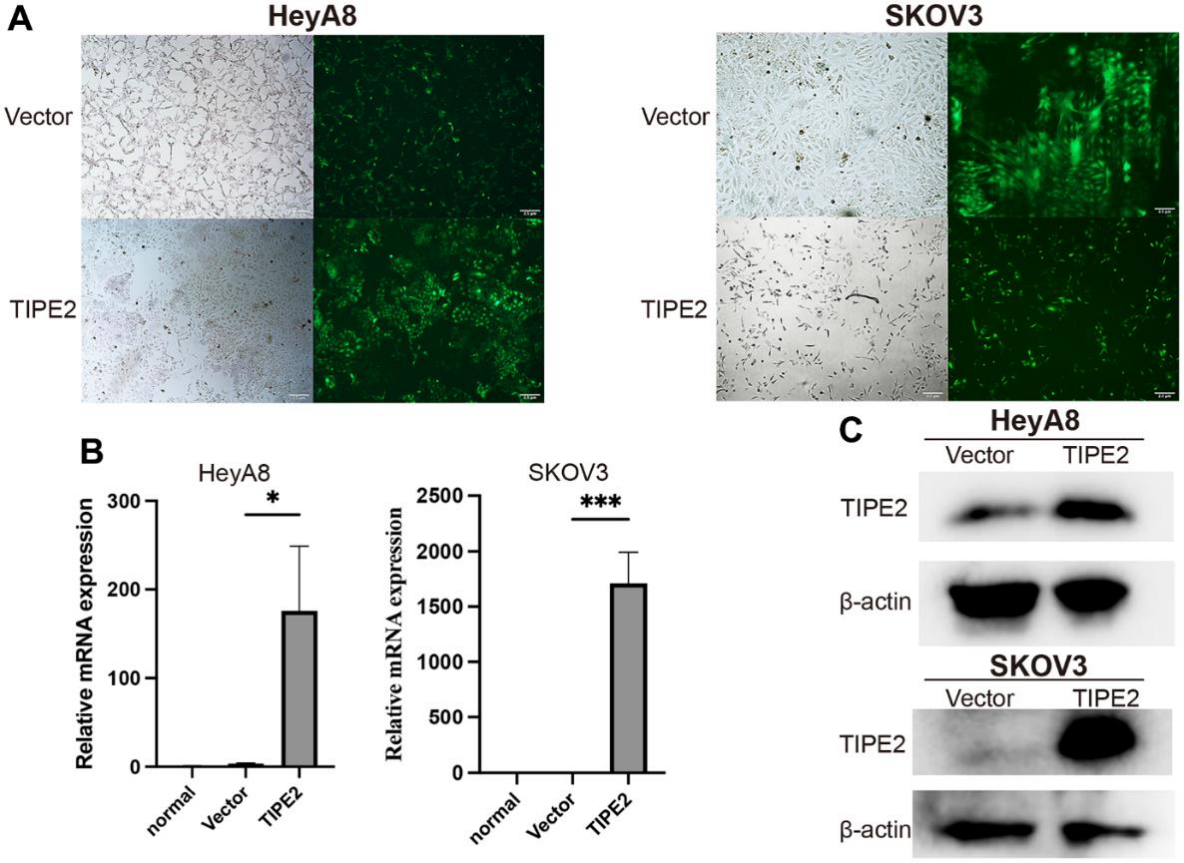
**Overexpression of TIPE2 in EOC cells by lentivirus transfection.** (**A**) Stable transfected EOC cell lines were established by lentivirus transfection. Representative images of stably transfected cells in bright field and dark fields are shown. Fluorescence microscopy showed that transfection efficiency was approximately 80%, and the cell state was normal. (**B**) qRT-PCR was conducted to compare endogenous TIPE2 expression between EOC cells transduced with lentivirus (n=3). TIPE2 expression was normalized to β-actin control. (**C**) TIPE2 protein expression in HeyA8 and SKOV3 cells by western blot analysis. β-actin was used as an internal control. Data are presented as mean ± SD. Statistical significance is indicated as: *p < 0.05, ***p < 0.001.

To further investigate TIPE2’s biological activity in EOC cells, lentivirus-mediated transfection was used to establish TIPE2 overexpression HeyA8, SKOV3, and control vector cell lines. The preliminary studies indicated that the basal expression of TIPE2 in these two EOC cell lines was lower. Following transduction for 72h, fluorescence microscopy observation was performed to assess green fluorescence protein (GFP) expression, and the results showed that the cell state was normal (Figure 2A). TIPE2 mRNA and protein levels were measured using qRT-PCR and Western blot following transfection. As indicated in Figure 2B and 2C, the levels in EOC cells transfected with TIPE2 were obviously upregulated compared with vector controls, and the difference was statistically significant.

**Figure 3:**
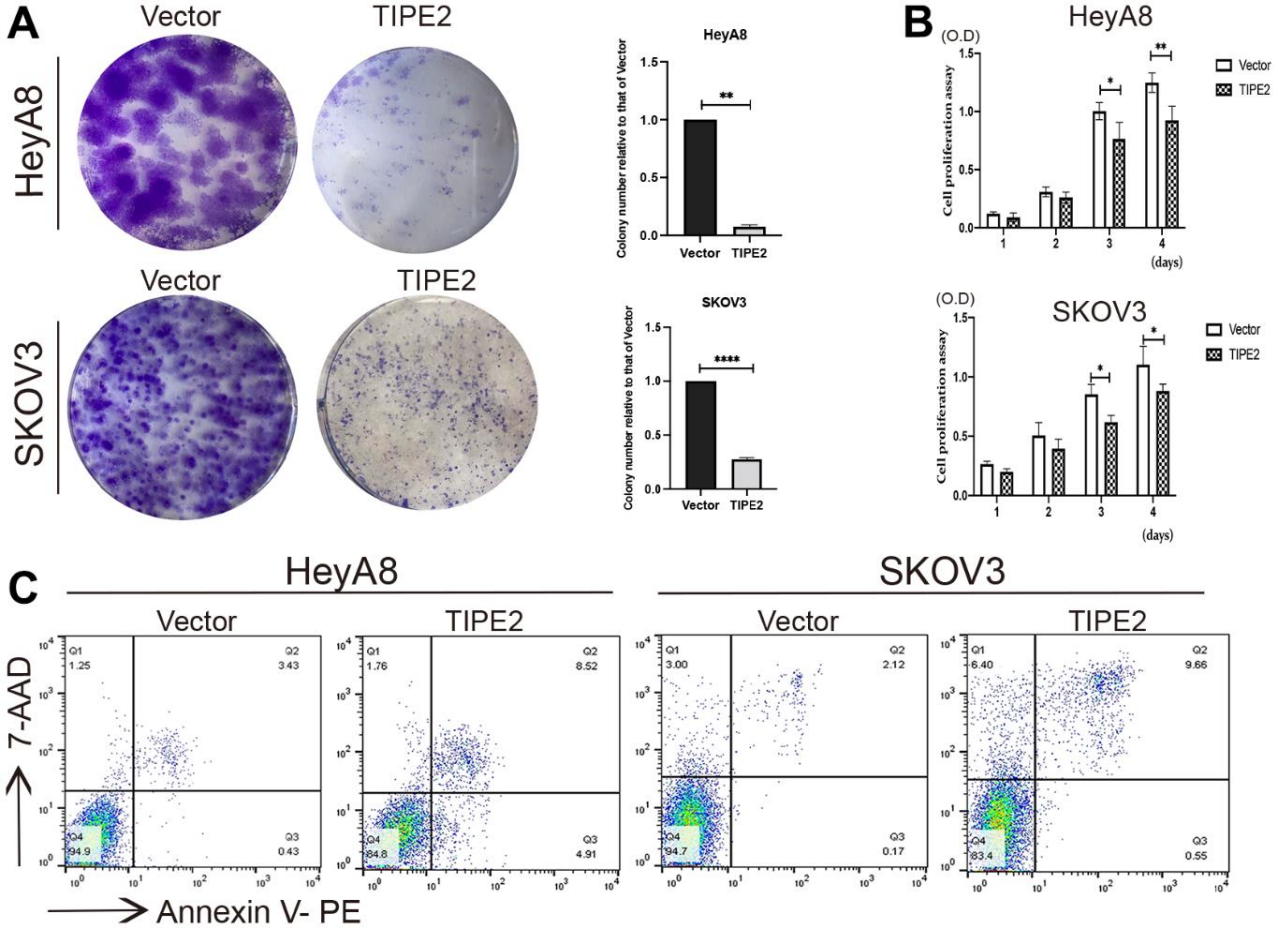
**Overexpression of TIPE2 suppresses ovarian cancer cell growth and increased cell apoptosis.** (**A**) Imagines of colony formation. 1×10^3^ HeyA8 and SKOV3 cells transfected with TIPE2, and its control plasmid mock were cultured to a new well with medium containing 10% FBS serum, and cell colony formation was examined by staining with crystal violet (n=3). (**B**) Cell viability of HeyA8 and SKOV3 cells with stable expression of TIPE2 or vector control was also determined by CCK8 assay (n=3). (**C**) Cell apoptosis was detected using flow cytometry. (Annexin V+/7-AAD-, early apoptosis; Annexin V+/7-AAD+, late apoptosis). Data are presented as mean ± SD. Statistical significance is indicated as: *p < 0.05, **p < 0.01, ****p <0.0001.

To further explore the effect of TIPE2 on EOC cell proliferation *in vitro*, HeyA8, and SKOV3 cells were evaluated for growth changes following TIPE2 transfection. Figure 3A demonstrates that TIPE2 dramatically inhibited the clonogenic capacity of HeyA8 and SKOV3 cells compared to the control cells. Then we performed a CCK8 assay and found that TIPE2 overexpression considerably reduced the ability of EOC cells to proliferate in a time-dependent manner by detecting the OD value matching the cell growth curve with a microplate reader (Figure 3B). Following that, flow cytometry was utilized to investigate the effect of TIPE2 on apoptosis in HeyA8 and SKOV3 cells using Annexin V-PE/7-AAD double labeling. Figure 3C shows a dramatically higher apoptosis rate in the HeyA8 cells expressing TIPE2 (13.43%) as compared to the vector group (3.86%). Similar results were obtained in SKVO3 cells (10.21% vs. 2.29%), indicating that TIPE2 significantly boosted cell apoptosis, through which it slowed down the proliferation of EOC cells *in vitro*.

### TIPE2 suppresses the mobility of EOC cells *in vitro*

**Figure 4:**
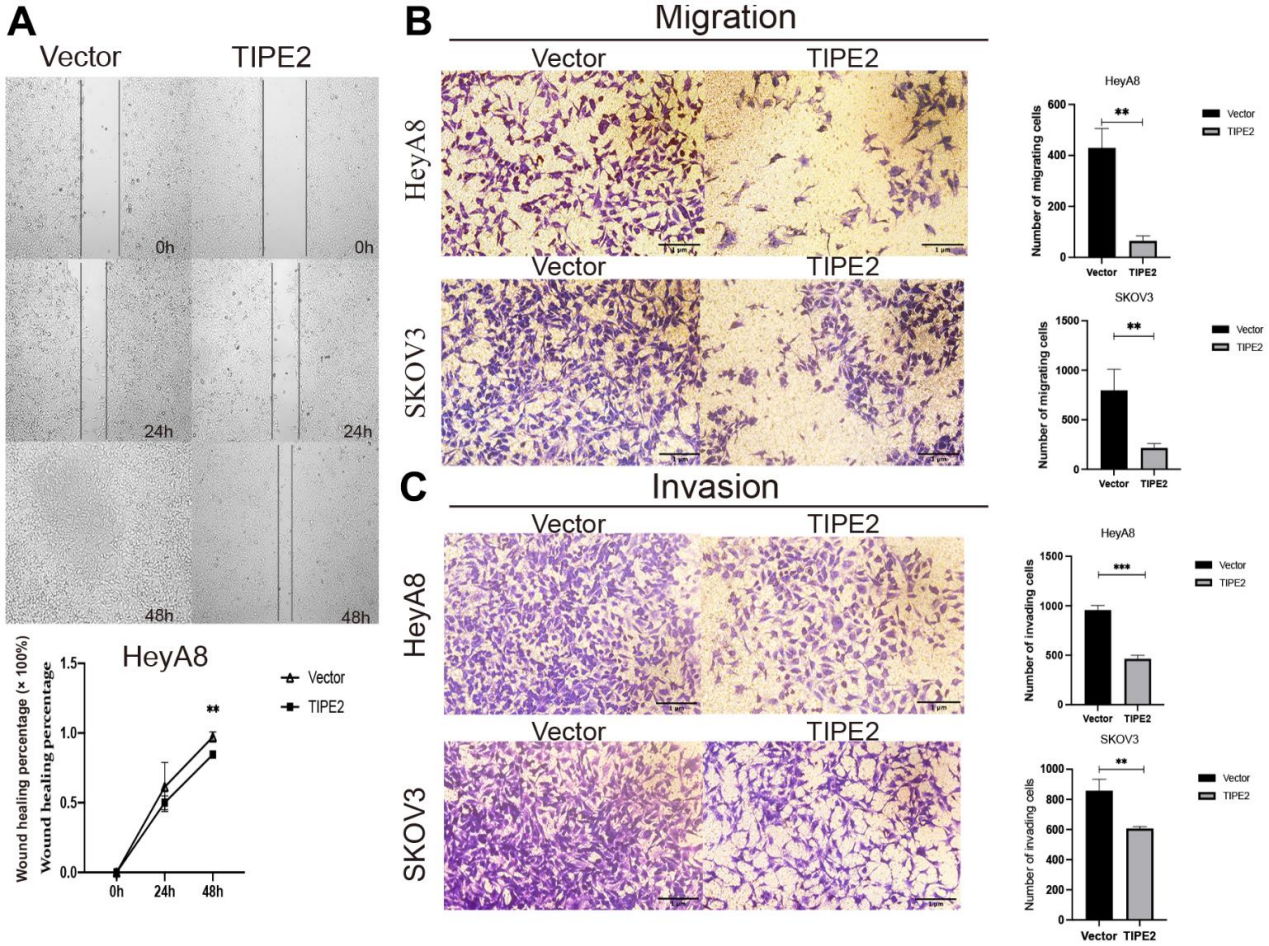
**Overexpression of TIPE2 suppresses the migration and invasion of EOC cells.** (**A**) HeyA8 cell migration was evaluated by the wound-healing assay using Culture-Inserts for Live Cell Analysis. Cells were seeded in culture inserts with a concentration of 3×10^5/^ml. After cells had grown to a dense cell layer overnight, inserts were carefully peeled off, resulting in a gap of 500 μm between the cell population. The wounded cell layer was photographed at times 0, 24, and 48h under the microscope. The invasive capability was determined by measuring the reduction in wound area between 0, 24, and 48h in HeyA8 cells (n=3). (**B**) Inhibition of cell migration in transwell assay. HeyA8 and SKOV3 cells transfected with TIPE2, and its control group was added to the upper chamber of a 24-well transwell. After incubation for 24 hours, the tumor cells on the bottom side of the chamber were fixed and stained with crystal violet (n=3). (**C**) Inhibition of cell invasion in a transwell assay. HeyA8 and SKOV3 cells transfected with TIPE2, and its control group were incubated for 48 hours in the upper chamber with Matrigel transwell filters from which cancer cells can invade into the lower chamber. Cells on the surface of the lower chamber were fixed and stained with crystal violet (n=3). Data are presented as mean ± SD. Statistical significance is indicated as: **p < 0.01, ***p < 0.001.

Metastasis is an important feature of cancer. To excavate the potential feature of TIPE2 in EOC mobility, the wound healing assay was performed in HeyA8 cells using Culture-Inserts for Live Cell Analysis (Ibidi, Martinsried, Germany), and cells migrating into the gap were tracked within 48h. By 24 hours, the wounds were apparent and the rate at which TIPE2 overexpressing cells migrated toward the wound was almost the same as that of the vector group. By 48 hours, however, the incision of the HeyA8/Vector group was nearly completely healed (Figure 4A). As expected, the wound healing assay uncovered that upregulation of TIPE2 significantly inhibited the migratory ability of EOC cells. Transwell assay was employed to further investigate the effect of TIPE2 on EOC cell migratory and invasive capacities *in vitro*. Consistently, the metastatic ability of HeyA8 and SKOV3 cells were all obviously suppressed after TIPE2 overexpression (Figure 4B, 4C). These findings imply that TIPE2 reduces the migration and invasion ability of EOC cells *in vitro*.

### PI3K/Akt signaling pathway mediates the anti-tumor function of TIPE2 in EOC cells

**Figure 5:**
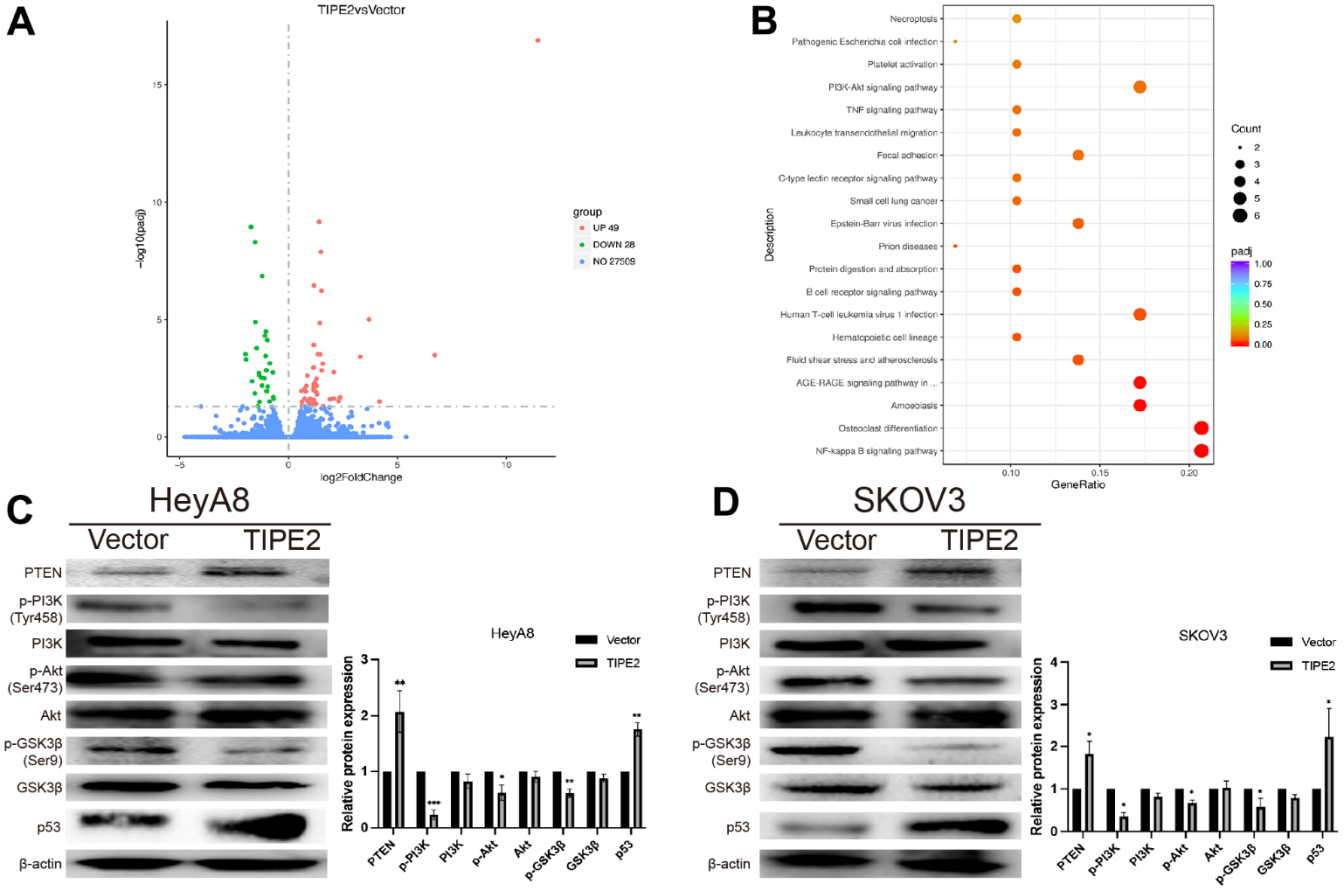
**TIPE2 suppresses the progression of EOC via inhibition of the PI3K/Akt/GSK3β signaling pathways.** (**A**) 49 upregulated and 28 downregulated genes in the SKOV3/TIPE2 group compared with the vector group on |Foldchange| > 0 and adjusted p < 0.05. (**B**) KEGG pathway annotation of differentially expressed genes. (**C**, **D**) Western blot analysis of PTEN, p-PI3K, PI3K, p-AKT, AKT, p-GSK3β, GSK3β and p53. The whole lysates derived from TIPE2 and vector infected HeyA8 and SKOV3 human EOC cells were immunoblotted with a panel of antibodies specific for PTEN, p-PI3K (Tyr458), PI3K, p-Akt (Ser473), Akt, p-GSK3β (Ser9), GSK3β, p53 and β-actin (a loading control) (n=3). Data are presented as mean ± SD. Statistical significance is indicated as: *p < 0.05, **p < 0.01, ***p < 0.001.

To further elucidate the molecular mechanism by which TIPE2 suppresses the malignancy of EOC cells, the RNA-seq based transcriptome analysis was conducted in TIPE2 overexpressing SKOV3 cells and control cells. The differentially expressed genes (DEGs) volcano plots are represented in Figure 5A. There were 49 upregulated and 28 downregulated genes in the SKOV3/TIPE2 group compared to the vector group, as determined by |log2Foldchange| > 0 and p < 0.05. The biological functions of DEGs were then determined using the Kyoto Encyclopedia of Genes and Genomes (KEGG), which indicated that the biological processes were enriched in the NF-kappa B signaling system, osteoclast differentiation, amoebiasis, and other pathways (Figure 5B). Notably, the PI3K/Akt signaling pathway, a well-known activated intracellular signaling pathway in various human malignancies, was suppressed by TIPE2 overexpression. Western blot analysis was carried out to determine the expression levels of PI3K/Akt signaling pathway-related proteins. The results showed that overexpression of TIPE2 reduced the phosphorylation levels of PI3K, Akt, and the downstream effector molecule GSK3β in HeyA8 and SKOV3 cell lines, whereas the total protein level did not change appreciably (Figure 5C, 5D). Overexpression of TIPE2 also increased the PTEN levels in EOC cell lines, which is a negative regulator of the PI3K/Akt signaling pathway, and its suppression would lead to constitutive activation of Akt [36, 37]. These findings imply that TIPE2 may exert its anti-tumor effect by inhibiting the PI3K/Akt signaling pathway. P53 inhibition is a crucial event in tumor development [38]. Western blot analysis revealed that overexpression of TIPE2 also increased the expression of p53 in HeyA8 and SKOV3 cell lines (Figure 5C, 5D).

### Effects of TIPE2 overexpression combined with PI3K pathway activation on the phenotypes of EOC cells

**Figure 6:**
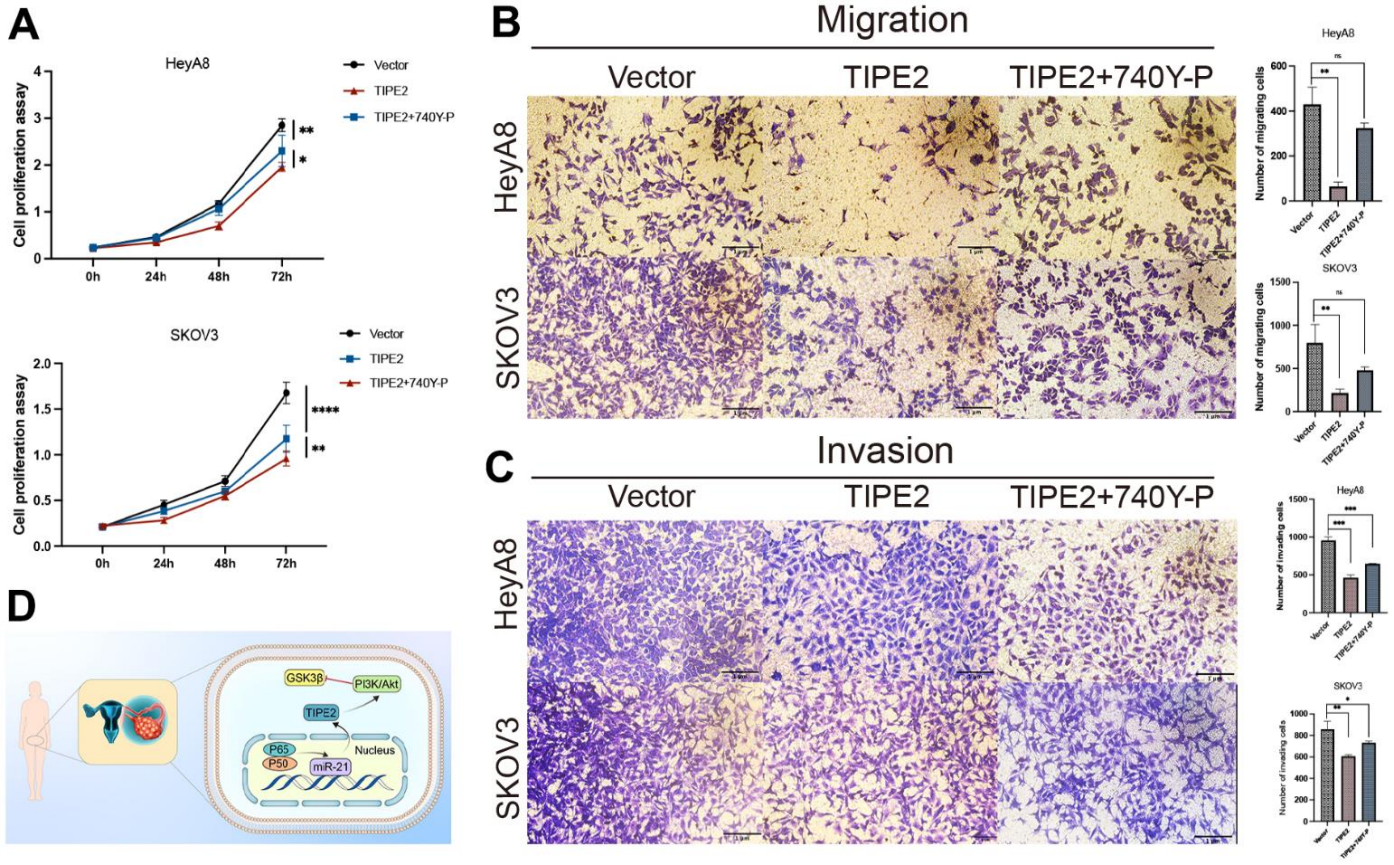
**740Y-P partially antagonized the inhibitory effect of TIPE2 on EOC cells.** (**A**) HeyA8 and SKOV3 cells with stable expression of TIPE2 were incubated with the PI3K agonist 740Y-P. CCK8 assay confirmed that the PI3K agonist 740Y-P could partially reverse the antiproliferation effect of TIPE2 (n=3). (**B**, **C**) Transwell migration assay and transwell invasion assay results confirmed that the PI3K agonist 740Y-P can partially reverse the anti-migration and anti-invasion effects of TIPE2 on EOC cells (n=3). (**D**) Schematic diagram of the anti-tumor activity of TIPE2 in human EOC. Data are presented as mean ± SD. Statistical significance is indicated as: * *p* < 0.05, ** *p* < 0.01, *** *p* < 0.001.

The relationship between TIPE2 and the PI3K/Akt signaling pathway was further investigated by treating TIPE2 transfected HeyA8 and SKOV3 cells with 740Y-P, a nonspecific agonist of PI3K [39]. TIPE2 overexpression-induced proliferation suppression of HeyA8 and SKOV3 cells was partially restored by pretreatment with 740Y-P, as shown in Figure 6A. Furthermore, co-treatment with 740Y-P dramatically reversed TIPE2-induced suppression of EOC cell migration and invasion (Figure 6B, 6C). The schematic diagram of the anti-tumor activity of TIPE2 in human EOC is shown in Figure 6D [40].

### TIPE2 expression is correlated with immune cell infiltration in ovarian cancer

**Figure 7:**
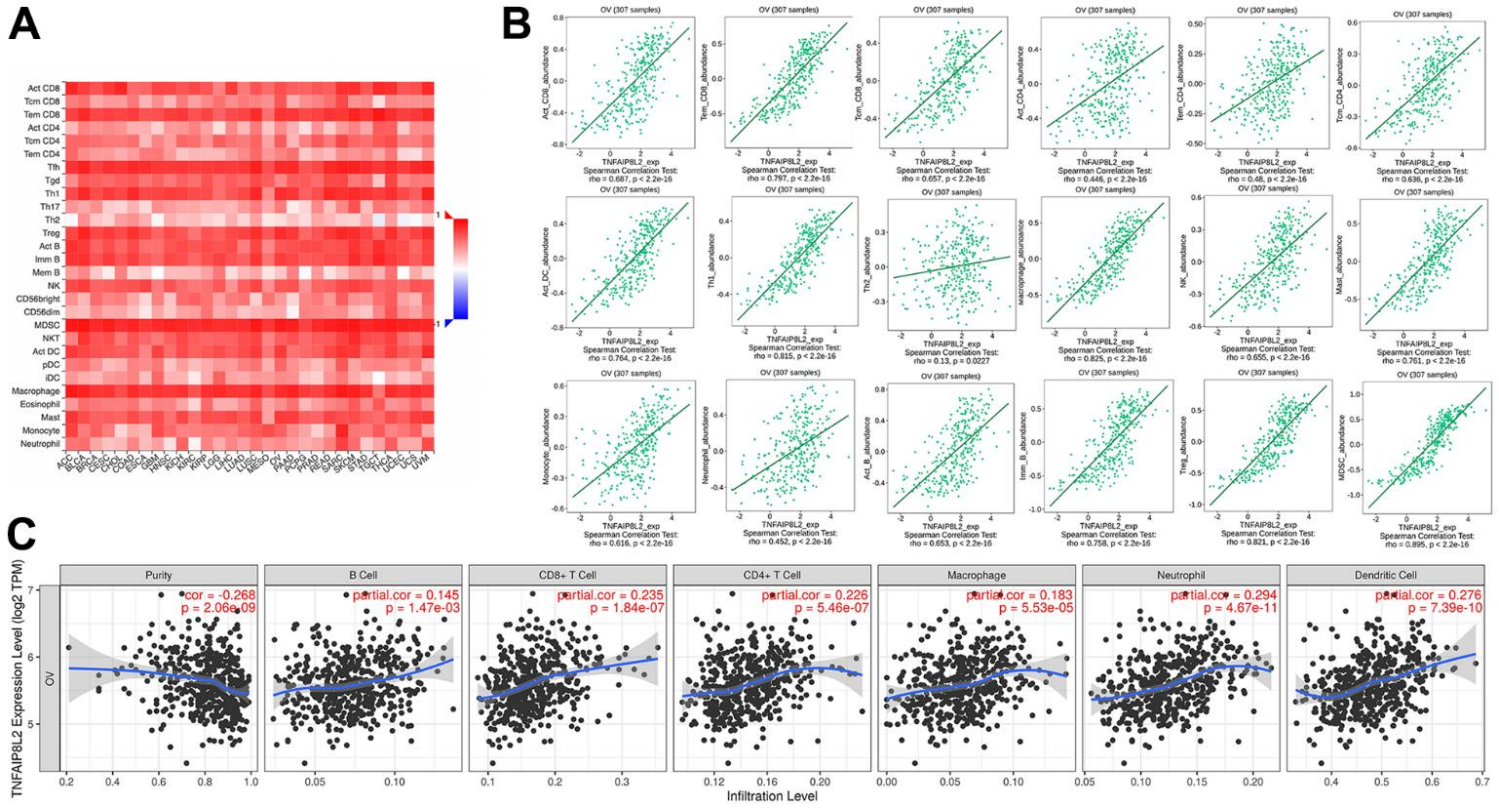
**The expression of TIPE2 is correlated with immune infiltration in ovarian cancer.** (**A**) Correlation between the expression of TIPE2 and the abundance of 28 types of tumor-infiltrating immune cells across human cancers available at TISIDB database. (**B**) The correlation between the expression of TIPE2 and the abundance of different immune cells in ovarian cancer on the TISIDB database. (**C**) TIPE2 expression is negatively related to tumor purity, and the expression of TIPE2 expression was correlated with infiltration levels of B cell, CD8+ T cell, CD4+ T cell, macrophage, neutrophil, and dendritic cell in ovarian cancer available at TIMER database.

Previous research has demonstrated that the presence of inflammatory cells in the tumor microenvironment of various solid tumors is associated with patient outcome, independent of tumor characteristics and that it may be used as a standalone indicator for predicting the stage and prognosis of cancer [41]. TIPE2 is a crucial regulator of immunological homeostasis, thus TISIDB and TIMER platforms were utilized to further explore the relationship between TIPE2 and TME in ovarian cancer. As shown in Figure 7A, 7B, significant correlations were observed between TIPE2 expression and immune cell infiltration using the TISIDB database, including activated CD8 T cells (Act_CD8, rho=0.687, *p* <2.2e-16), effector memory CD8 T cells (Tem_CD8, rho=0.797, *p* <2.2e-16), central memory CD8 T cells (Tcm_CD8, rho=0.657, *p* <2.2e-16), natural killer cells (NK, rho=0.655, *p* <2.2e-16), type 1 T helper cells (Th1, rho=0.815, *p* <2.2e-16), macrophages (rho=0.825, *p* <2.2e-16), activated dendritic cells (act_DC, rho=0.764, *p* <2.2e-16), mast cells (Mast, rho=0.761, *p* <2.2e-16), and immature B cells (Imm_B, rho=0.758, *p* <2.2e-16). The correlation between TIPE2 and immune cell infiltration was also confirmed by the TIMER tool (Figure 7C). We further performed correlation analysis between TIPE2 expression and biomarkers expressing in distinct immune cells (Tables 2, 3). Significant correlations were observed between TIPE2 expression and CD8+ T cells, T cell exhaustion (immune checkpoint genes), general T cells, Th1, Th2, Tfh, Treg, B cells, monocyte, M1 macrophages, M2 macrophages, neutrophils, NK cells in ovarian cancer. TIPE2 was shown to be associated with tumor-infiltrating immune cells in the TME of ovarian cancer.

**Table 2:** Correlation analysis between TIPE2 and related genes and markers of immune cells in Tumor Immune Estimation Resource (TIMER).

Description	Gene markers	None		Purity	
		Cor	p	Cor	p
CD8+ T cell	CD8A	0.349	***	0.474	***
	CD8B	0.52	***	0.355	***
	GZMK	0.725	***	0.566	***
T cell (general)	CD2	0.766	***	0.626	***
	CD3D	0.732	***	0.572	***
	CD3E	0.734	***	0.567	***
B cell	CD19	0.069	0.231	-0.003	0.961
	CD38	0.538	***	0.469	***
	CD79A	0.436	***	0.239	**
Monocyte	CD86	0.912	***	0.863	***
	CSF1R	0.881	***	0.817	***
TAM	CCL2	0.616	***	0.447	***
	CD68	0.857	***	0.78	***
	IL10	0.54	***	0.351	***
M1 macrophage	IRF5	0.421	***	0.413	***
	TLR2	0.763	***	0.651	***
	TLR4	0.84	***	0.74	***
	CD80	0.674	***	0.602	***
	FCGR1A	0.907	***	0.862	***
	NOS2	-0.014	0.804	-0.147	0.021
	PTGS2	0.159	*	-0.025	0.697
M2 macrophage	CD163	0.783	***	0.673	***
	MS4A4A	0.812	***	0.699	***
	VSIG4	0.809	***	0.706	***
	CD115	0.881	***	0.817	***
	CD206	0.551	***	0.352	***
	CXCR1	0.264	***	0.113	0.0744
	CXCR4	0.08	0.163	0.044	0.490
Neutrophil	CCR7	0.649	***	0.482	***
	ITGAM	0.822	***	0.741	***
	CEACAM8	-0.073	0.206	-0.05	0.431
	FCGR3B	0.408	***	0.236	**
	SELL	0.623	***	0.462	***
NK cell	KIR3DL1	0.343	***	0.228	**
	KIR3DL2	0.247	***	0.102	0.107
	KIR3DL3	0.118	0.040	0.05	0.436
	KIR2DL1	0.197	**	0.066	0.298
	KIR2DL3	0.255	***	0.202	*
	KIR2DL4	0.478	***	0.356	***
	KIR2DS4	0.241	***	0.134	0.0349
	GNLY	0.542	***	0.443	***
	PRF1	0.689	***	0.531	***
Dendritic cell	HLA-DPA1	0.754	***	0.658	***
	HLA-DPB1	0.779	***	0.674	***
	HLA-DRA	0.733	***	0.649	***
	HLA-DQB1	0.557	***	0.436	***
	CD1C	0.624	***	0.488	***
	ITGAX	0.799	***	0.702	***
	NRP1	0.41	***	0.166	*
Th1	CXCR3	0.739	***	0.604	***
	STAT1	0.224	***	0.198	*
	STAT4	0.622	***	0.442	***
	TBX21	0.707	***	0.551	***
	TNF (TNF-α)	0.372	***	0.264	***
	IFNG (IFN-γ)	0.541	***	0.383	***
Th2	GATA3	0.424	***	0.152	0.017
	IL13	0.146	0.011	0.128	0.043
	STAT6	0.096	0.096	0.096	0.13
	STAT5A	0.35	***	0.371	***
Tfh	BCL6	0.048	0.407	0.141	0.027
	IL21	0.237	***	0.267	***
Th17	STAT3	0.254	***	0.194	*
	IL17A	0.147	0.010	0.063	0.322
Treg	CCR8	0.499	***	0.381	***
	FOXP3	0.624	***	0.468	***
	TGFB1 (TGFβ)	0.625	***	0.388	***
T cell exhaustion	CTLA4	0.674	***	0.518	***
	HAVCR2 (TIM3)	0.919	***	0.865	***
	LAG3	0.576	***	0.513	***
	GZMB	0.52	***	0.336	***
	PDCD1 (PD-1)	0.577	***	0.443	***

Note. Cor, R value of Spearman’s correlation; None, correlation without adjustment; Purity: Correlation adjusted by purity. *p < 0.01; **p < 0.001; ***p < 0.0001.

**Table 3:** Correlation analysis between TIPE2 and related genes and markers of immune cells in Gene Expression Profiling Interaction Analysis (GEPIA).

Description	Gene markers	None	
		Cor	p
CD8+ T cell	CD8A	0.69	***
	CD8B	0.61	***
	GZMK	0.7	***
T cell (general)	CD2	0.81	***
	CD3D	0.74	***
	CD3E	0.78	***
B cell	CD19	-0.037	0.4
	CD38	0.67	***
	CD79A	0.5	***
Monocyte	CD86	0.92	***
	CSF1R	0.89	***
		
TAM	CCL2	0.67	***
	CD68	0.84	***
	IL10	0.63	***
M1 macrophage	IRF5	0.5	***
	TLR2	0.74	***
	TLR4	0.45	***
	CD80	0.58	***
	FCGR1A	0.86	***
	NOS2	-0.16	**
	PTGS2	0.071	0.11
M2 macrophage	CD163	0.61	***
	MS4A4A	0.74	***
	VSIG4	0.82	***
	CD115	0.83	***
	CD206	0.25	***
	CXCR1	0.15	**
	CXCR4	0.2	***
Neutrophil	CCR7	0.42	***
	ITGAM	0.77	***
	CEACAM8	-0.015	0.73
	FCGR3B	0.64	***
	SELL	0.72	***
NK cell	KIR3DL1	0.47	***
	KIR3DL2	0.5	***
	KIR3DL3	0.23	***
	KIR2DL1	0.37	***
	KIR2DL3	0.5	***
	KIR2DL4	0.6	***
	KIR2DS4	0.43	***
	GNLY	0.59	***
	PRF1	0.74	***
Dendritic cell	HLA-DPA1	0.8	***
	HLA-DPB1	0.82	***
	HLA-DRA	0.77	***
	HLA-DQB1	0.61	***
	CD1C	0.68	***
	ITGAX	0.75	***
	NRP1	0.11	0.011
Th1	CXCR3	0.8	***
	STAT1	0.5	***
	STAT4	0.31	***
	TBX21	0.66	***
	TNF (TNF-α)	0.53	***
	IFNG (IFN-γ)	0.54	***
Th2	GATA3	0.54	***
	IL13	-0.073	0.099
	STAT6	-0.13	*
	STAT5A	0.089	0.045
Tfh	BCL6	-0.046	0.3
	IL21	0.36	***
Th17	STAT3	0.15	**
	IL17A	0.15	**
Treg	CCR8	0.58	***
	FOXP3	0.7	***
	TGFB1 (TGFβ)	0.64	***
T cell exhaustion	CTLA4	0.7	***
	HAVCR2 (TIM3)	0.92	***
	LAG3	0.11	0.014
	GZMB	0.63	***
	PDCD1 (PD-1)	0.66	***

Note. Tumor, correlation analysis in normal tissue of TCGA. *p < 0.01; **p < 0.001; ***p < 0.0001.

**Figure 8:**
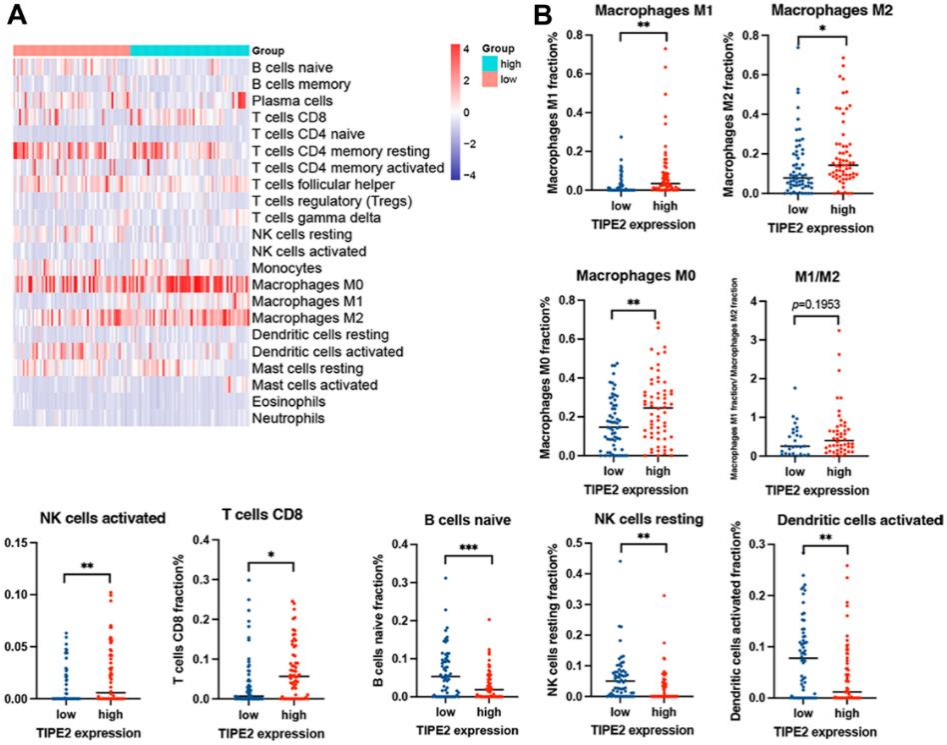
**Analysis of the GEO dataset using CIBERSORT online.** (**A**) The abundance of tumor-infiltrating immune cells via the LM22 signature matrix in ovarian cancer. (**B**) Tumor-infiltrating immune cells were plotted according to the TIPE2 expression level. Statistical significance is indicated as * *p* < 0.05, ** *p* < 0.01, *** *p* < 0.001.

The CIBERSORT was then utilized to further quantify the proportion of immune cells in ovarian cancer with low and high TIPE2 expression. We downloaded two mRNA expression datasets (GSE51373 (n=28), and GSE103737 (n=97)) from the Gene Expression Omnibus (GEO) and tumor-infiltrating immune cells were calculated using the CIBERSORT algorithm (Figure 8A). We divided the samples in each dataset into two groups according to TIPE2 expression and analyzed the tumor-infiltrating immune cells between patients with low and high TIPE2 expression. The results showed that TIPE2 expression had a positive correlation with immune cells including M0, M1, M2 macrophages, NK cells activated, and CD8+ T cells, while the levels of B cells naïve, NK cells resting, and dendritic cells activated were dramatically downregulated in the group with high expression of TIPE2 (Figure 8B). Furthermore, the expression levels of TIPE2 were highly linked with M1 macrophage, and positively correlated with the M1/M2 ratio, although this is not statistically significant. The results indicated that macrophages tend to polarize towards the M1 phenotype with high TIPE2 expression. Tumor-associated macrophages (TAMs) are the most abundant infiltrative immune cells in and around tumors, contributing to tumor proliferation, angiogenesis, and therapy resistance [42]. TIPE2 may participate in TAM polarization towards an M1 phenotype and play anti-tumoral functions in the TME of ovarian cancer, which must be explored further.

## DISCUSSION

The incidence and mortality of cancer are increasing globally, largely due to population growth and aging [2]. Inflammation mediators are essential for the maintenance of physiological homeostasis. Rudolf Virchow first proposed the function of inflammation in cancer in 1863 after observing the presence of leukocytes in tumor tissue. Currently, around 25% of cancers are etiologically linked to infectious disease and chronic inflammation [43–45]. In recent years, TME has received much attention. Chemokines, cytokines, and small inflammatory proteins produced by either malignant or host cells (i.e., inflammatory cells, stromal, and endothelial cells) cooperate in the TME, and this crosstalk between cells and associated cytokines is essential for tumor growth and metastasis [46]. On the other hand, systemic inflammation including cytokines, immune cells, and small inflammatory proteins are crucial to the dissemination of tumor [47]. Some of the mechanisms underlying the relationship between inflammation and cancer have been clarified. As an example, the NF-κB family has been noted to be important for both cancer and innate immunity [48]. Activation of NF-κB is part of the immune defense that could identify and eliminate the transformed cells. Furthermore, the NF-κB signaling pathway is active in several tumor types exerting its cancer-promoting effects. It also cooperates with several important signaling molecules like GSK3β, which is recognized as a modulator of many critical signaling pathways including the PI3K, Notch signaling, and the Wnt signaling pathways [49]. Targeting cancer-related inflammation has the potential to favorably affect both the local TME and systemic circulation, and thus benefit the patient [17].

In this study, we first discovered that TIPE2 expression was downregulated in both EOC tissues and cell lines, which is inconsistent with previous studies, demonstrating the possible TIPE’s suppressive role in EOC [50]. Further experiments were conducted to investigate the role of TIPE2 in the development and progression of EOC, and our results indicated that stable overexpression of TIPE2 inhibits the pathological processes, such as proliferation, invasion, metastasis, and promotes the apoptosis of EOC cells.

Then the RNA-seq-based transcriptome analysis was utilized to elucidate the detailed molecular mechanism by which TIPE2 exerts its anti-tumor effects in EOC. PI3K/Akt signaling pathway was selected from all the DEGs, and the results showed that overexpression of TIPE2 drastically reduced the phosphorylation levels of PI3K and Akt in EOC cells. Furthermore, TIPE2 overexpression-induced proliferation and metastatic suppression in EOC cells could be partially restored by pretreatment with 740Y-P, a nonspecific agonist of PI3K. PI3K/Akt signaling pathway is the most frequently activated intracellular signaling pathway in diverse human malignancies, which plays a vital role in the unrestricted proliferation, metabolic programming, autophagy, transcription, and angiogenesis of cancer [51, 52]. According to TCGA data, the PI3K/Akt signaling pathway is overstimulated in about 60% of ovarian cancers [53]. It is being investigated as a potential therapeutic target as it has been shown to be associated with resistance to chemotherapy and radiotherapy of ovarian cancer [54, 55].

Akt has several targets, the majority of which are transcription factors that may influence cancer cell metabolism and function. Research indicates that Akt activation can directly phosphorylate GSK3 at Ser9 and suppress its kinase activity, thereby reducing the expression level of GSK3β [56, 57]. GSK3 has two isoforms in mammals: GSK3α and GSK3β [58]. GSK3 is a potential therapeutic target for cancers, and inhibiting GSK3 would promote tumor progression by stimulating cell proliferation and substantially decreasing the number of apoptotic cells. Recent studies suggest that GSK3 also appears to be critical in the inflammatory process. Overexpression of TIPE2 may stimulate NF-κB mediated transcription, which is associated with many inflammatory signaling cascades [59]. By decreasing the cytokines generated by monocytes, GSK3 inhibition could prevent the detrimental effects of acute inflammation on tumor growth [60]. Western blot confirmed that the inhibition of the PI3K/Akt signaling pathway in TIPE2 overexpressing EOC cells would lead to the activation of GSK3β. Additionally, PTEN was found to be upregulated by TIPE2 overexpression, which is an essential tumor-suppressing gene and a prominent negative regulator of PI3K/Akt signaling pathway. The loss of its function is regarded as a founding genetic event in carcinogenesis [61]. Collectively, TIPE2 may act as a tumor suppressor in EOC through PTEN activation and PI3K/Akt signal inactivation, which in turn leads to GSK3β dephosphorylation.

TME is comprised of dividing tumor cells, immune cells, endothelial cells, fibroblasts, blood vessels, and metabolites [62–64]. Ovarian cancer is classified as an immunoreactive tumor [65, 66]. Recently, the critical role of TME in the development and progression of ovarian cancer, and in the resistance against antitumor therapy has received increasing attention. Although the essential processes underlying the interaction between inflammation and tumor-host immunity remain a mystery. In recent years, chimeric antigen receptor- and TCR-engineered T cells, immune checkpoint inhibitors (including CTLA-4 and PD-1/PD-L1 inhibitors), cancer vaccines, and oncolytic viruses which would reverse the signals from the TME have been investigated as potential therapies for ovarian cancer [67]. TIPE2 essentially regulates both innate and adaptive immune responses, and loss of TIPE2 was found to induce systemic autoimmunity in humans and fatal inflammatory diseases in mice [20]. Research showed that TIPE2 can regulate tumorigenesis not just directly from the interior of tumor cells, but also indirectly through immune cells in pancreatic cancer [68]. It’s analogous to altering both the seed and the soil environment. With the help of the online database, we further explore the link between TIPE2 and immune infiltration in ovarian cancer. The results showed that TIPE2 expression correlated positively with the tumor-infiltrating immune cells in ovarian cancer, including CD8+ T cells, CD4+ T cells, Treg cells, activated DCs, NK cells, macrophages, mast cells, and Tfh, suggesting that TIPE2 may impair the TME of ovarian cancer. TIPE2 expression also showed a significant association with immune cell markers like T cell exhaustion markers (e.g., CTLA4, HAVCR2, GZMB, PDCD1, FOXP3, and CCR8). T cell exhaustion is a hallmark of cancer that refers to a condition in which T cells are dysfunctional [69]. Elucidating the molecular mechanism of T-cell exhaustion is a key objective in cancer immunotherapy and reversing this through checkpoint blockade is remarkably effective in cancer treatment [70].

The CIBERSORT was then utilized to further quantify the proportion of immune cells in ovarian cancer with low and high TIPE2 expression. We downloaded two mRNA expression datasets from the GEO and tumor-infiltrating immune cells were calculated using the CIBERSORT algorithm. Our results demonstrated that the expression levels of TIPE2 showed remarkably higher correlation with M1 macrophages, and positively correlated with the M1/M2 ratio, albeit this was not statistically significant. Macrophages with high TIPE2 expression tend to polarize towards the M1 phenotype. The regulation between TAMs and ovarian cancer cells is bidirectional, with M1 macrophages tending to be pro-inflammatory with anti-tumor activity, while M2 macrophages possess anti-inflammatory traits that may promote tumor progression [71]. TAMs are ideal targets for oncotherapy either by redifferentiation from pro-tumor towards anti-tumor states or by reducing macrophage infiltration in the tumor [72].

According to research, those with upregulated M1/M2 ratios had considerably improved 5-year survival rates than those with low counts [73]. Therefore, a TIPE2-stimulated co-culture system of ovarian cancer cells and macrophages may be conducted to further investigate whether TIPE2 can drive macrophage polarization in the TME of ovarian cancer. Taken together, our findings suggest that TIPE2 may play a role in ovarian cancer immunology and that TIPE2 might be exploited as a novel immunotherapy target for ovarian cancer.

## CONCLUSIONS

Numerous studies indicate that TIPE2 regulates tumorigenesis not just directly from inside tumor cells, but also indirectly through immune cells [74]. The present investigation reports for the first time that TIPE2 was decreased in EOC. Overexpression of TIPE2 inhibited the proliferation, metastasis, and promoted apoptosis of EOC cells. Mechanically, TIPE2 may act as a tumor suppressor by blocking the PI3K/Akt signaling pathway. Furthermore, TIPE2 was closely associated with the infiltration of TILs in ovarian cancer, lending credence to TIPE2’s putative immunological role in ovarian cancer. TIPE2 somehow functions as a biological link between cancer and inflammation. It urges us to explore the molecular foundations of TIPE2 as well as tumor-immune interactions in ovarian cancer in the future. TIPE2 has the potential to be employed as a therapeutic target and checkpoint in ovarian cancer.

## MATERIALS AND METHODS

### Patients tissue samples

Between January 2021 and July 2021, 12 EOC and 14 normal ovarian tissues were collected from surgical resections at the department of Gynecology, the Second Hospital of Shandong University (Jinan, Shandong, China). Tumor samples were extracted from patients with primary EOC and verified by operative pathology. Fourteen normal ovarian tissues were obtained from patients with benign diseases undergoing hysterectomy and bilateral salpingectomy. Patients with polycystic ovaries, ovarian cysts, or other complications were omitted. After isolation, the tissue specimens were immediately snap-frozen in liquid nitrogen and stored in a -80° C bio-freezer (Forma Scientific, USA) until further processing. The experimental design was sanctioned by the Second Hospital of Shandong University Ethics Committee (KYLL-2019(KJ)P-0060), and all the participants gave informed consent.

### The inclusion criteria:

Patients who didn’t receive any preoperative anticancer treatment, including chemo-, radio-, or immunotherapy.

Patients with no other coexisting malignancies.

### Cell lines and cell culture

In this investigation, four human EOC cell lines (A2780, OVCAR3, SKOV3, and HeyA8) and a human ovarian epithelial cell line (IOSE80) were purchased from the American Type Culture Collection (ATCC, Manassas, VA, USA). A2780, HeyA8, OVCAR3, and IOSE80 cells were regularly cultured in Dulbecco’s modified Eagle’s serum medium (Gibco), and SKOV3 were cultured in McCoy’s 5A medium (Gibco), supplemented with 10% fetal bovine serum (Invitrogen; Thermo Fisher Scientific, Inc., Waltham, MA, USA, USA) and 1% penicillin-streptomycin (Invitrogen; Thermo Fisher Scientific, Inc., USA). Cells were incubated at 37° C in a humidified incubator at 5% CO2/95% air environment.

### RNA extraction and quantitative real-time PCR (qRT-PCR)

Total RNA was extracted from biological specimens using Trizol (Invitrogen, Carlsbad, CA, USA) as directed by the manufacturer. The purity and concentration of RNA were determined using the Nanodrop 1000 spectrophotometer (Thermo Fisher Scientific). PrimeScript™ RT Reagent Kit (TaKaRa, Japan) was used to obtain reverse RNA, and the RNA was then transcribed to generate cDNA. SYBR Premix Ex TaqII Kit (TaKaRa, Japan) was used for qRT-PCR. The mRNA expression levels of TIPE2 were determined using TaqMan® gene expression assay in a 96-well plate format (442795, Applied Biosystems, USA). Gene expression levels were quantified in relation to the expression of β-actin which was employed as a loading control. Relative gene expression levels were normalized by log2 transformation. The PCR thermocycling settings were as follows: 95° C for 30 seconds; 40 amplification cycles of 95° C for 5 seconds, and 60° C for 30 seconds.

The sequence of TIPE2:

5’-GGAACTCCAAGGCAAGACTG-3’(forward),

5’-AGCACCTCACTGCTTGTCTCATC-3’(reverse).

The sequence of β-actin:

5’-GAAGAGCTACGAGCTGCCTGA-3’(forward),

5’-CAGACAGCACTGTGTTGGCG-3’(reverse).

### Western blot analysis

Proteins were extracted using RIPA lysis buffer (Beyotime Institute of Biotechnology, China) augmented with 1mmol/L phenylmethylsulphonyl fluoride (PMSF, Beyotime Institute of Biotechnology, China). The BCA (bicinchoninic acid) test was performed to quantify the concentration of the extracted protein. Protein samples were separated using SDS-PAGE and transferred to a PVDF membrane (Millipore, Billerica, MA, USA), followed by 1 hour of membrane blocking at room temperature with 5% non-fat milk in Tris-buffered saline and Tween-20 (TBST). Following blocking, the membranes were incubated overnight with primary antibodies at 4° C. Then the protein bands were incubated with secondary antibodies for 1 hour at room temperature and tested using an ECL system (Thermo Scientific Pierce).

The primary antibodies used are as follows: rabbit anti-human TIPE2 (1:1000 dilution; Abcam, Cambridge, MA, USA), anti-Akt (1:1000 dilution; Abcam, Cambridge, MA, USA), anti-phosphorylation of Akt (1:1000 dilution; Abcam, Cambridge, MA, USA), anti-PI3K (1:1000 dilution; Abcam, Cambridge, MA, USA), anti-phosphorylation of PI3K (1:1000 dilution; Abcam, Cambridge, MA, USA), anti-GSK3β (1:1000 dilution; Abcam, Cambridge, MA, USA), anti-phosphorylation of GSK3β (1:1000 dilution; Abcam, Cambridge, MA, USA), anti-p53 (1:1000 dilution; Abcam, Cambridge, MA, USA), anti-PTEN (1:1000 dilution; Abcam, Cambridge, MA, USA), and anti-β-actin polyclonal antibody (1:1000 dilution; Abcam, Cambridge, MA, USA) was used as a control. The secondary antibody was HRP-conjugated (1:3000, G1213-100UL, Servicebio Technology Co., Ltd., China).

### Endogenous overexpression of TIPE2 in EOC cell lines and treatment

The TIPE2 overexpression lentivirus vector- GV492 was constructed by Shanghai GeneChem Co., Ltd. (Shanghai, China). The sequences were as follows: Forward: AGGTCGACTCTAGAGGATCCCGCCACCATGGAGTCCTTCAGCTCAAAG; Reverse: TCCTTGTAGTCCATACCGAGCTTCCCTTCGTCTAGCAGCTTCCTG- AG. HeyA8 and SKOV3 cells were plated in a 24-well plate overnight for lentivirus transfection with a multiplicity of infection (MOI) of 10. The empty GV492 lentivirus vector serves as a control. To create stable TIPE2 overexpression cell lines, the cells were treated with 2μg/ml puromycin for 1 week. Western blot and qRT-PCR were used to evaluate the expression of TIPE2 after transfection. EOC cells transfected with TIPE2 were cultured for 48h in a medium containing 10 μl PI3K agonist (740Y-P, MCE, USA) for 48h. These cells were referred to as the TIPE2+ 740Y-P group.

### Cell proliferation assay

The cells’ capability to proliferate was evaluated by the Cell Counting Kit-8 assay (CCK-8; Dojindo, Kumamoto, Japan). Cells in the logarithmic phase were resuspended in full culture medium and seeded in a 96-well plate at a density of 2×103/100μl/well before cell adherence. At the indicated time point (24, 48, 72, and 96 hours), 10μl of CCK-8 solution was added to each well and incubated for another 3 hours. The optical density (OD) value at 450 nm was detected using a spectrophotometer (Thermo Fisher Scientific).

### Colony formation assay

EOC cells (1×10^3^/well) were laid on 6-well plates and cultured in medium augmented with 10% FBS serum. The medium was changed after cell adhesion and every 48 hours thereafter. Upon colony formation after 2 weeks, cells were washed with PBS buffer, fixed for 10 minutes using paraformaldehyde, and then stained for 20 minutes with crystal violet solution. The colony counting analysis was performed after thorough washing with PBS until no background Giemsa stain was visible.

### Wound healing assay

Wound healing assay was performed with the assistance of Culture-Inserts for live cell analysis (Ibidi, Martinsried, Germany). 70μl/well of cell suspensions were seeded into the culture inserts at a concentration of 3×105/ml. Following culture overnight, culture inserts were removed leaving a gap of 500μm between the cell population. Followed by PBS washing to remove cell debris, the cells were cultured in fresh medium and the growth pattern was photographed using a microscope within 48 hours.

### Transwell assays for cell migration and invasion

The impact of TIPE2 overexpression on ovarian cancer cell migration and invasion were evaluated via transwell assay (pore size 8 μm; Corning, New York, USA). For migration assays, 3×10^5^/well cells were resuspended in 200 μl serum-free medium and seeded into the upper chambers of 24-well transwell plates to migrate through a filter toward 10% serum-containing medium in the bottom chamber, ensuring that the bottom side of the membrane was in contact with the medium. The upper chamber membrane was pre-coated with 45 μl of Matrigel mix (BD Biosciences, San Diego, CA, USA) and incubated for 4-6 h at 37° C for invasion assay. The cells that went through the membrane after 24 hours for the migration experiment and 48 hours for the invasion assay were fixed with 4% paraformaldehyde for 10 minutes and stained with crystal violet solution for 20 minutes. Finally, stained cells were calculated from 5 randomly selected fields on the membrane via a light microscope at 200× magnification.

### Cell apoptosis detection

For the cell apoptosis assay, transfected HeyA8 and SKOV3 cells were inoculated into a 6-well plate, grown to 85% confluency, digested with trypsin, washed with PBS, centrifuged, and then reconstituted in 200 μl of 1× binding buffer. Then, the cells were stained with Annexin V-PE/ 7-AAD (Vazyme Biotech, China) for 20 min in light-resistant conditions according to the manufacturer’s instructions. Finally, flow cytometry was used to assess cell apoptosis and quantify the proportion of apoptotic cells.

### RNA-sequencing (RNA-seq) and data analysis

RNA-seq was used to analyze the gene expression profile in human SKOV3 cells overexpressing TIPE2 and control SKOV3 cells (Novogene Co., Ltd, Beijing, China). Total RNA was extracted using Trizol reagent (Invitrogen, Carlsbad, CA, USA), and each sample was replicated three times. The cDNA library was prepared according to the standard Illumina RNA-seq instructions, and basecalling was performed using Illumina Casava 1.7 software. Assembly of transcripts and estimation of their abundance (fragments per kilobase of exon per million fragments mapper, FPKM) were carried out using Cufflinks software. DESeq2 software was used to conduct differential gene expression analysis across groups with |log2Foldchange| > 0 and p < 0.05. All the raw data have been uploaded to the GEO database (GSE208351).

### Immune infiltration analysis of the TME

The Tumor Immune Single-cell Hub (TISCH) database, Tumor Immune Estimation Resource (TIMER) database, Tumor-Immune System Interaction (TISIDB) website, and the Gene Expression Profiling Interactive Analysis (GEPIA) online database were utilized to determine the correlation between TIPE2 expression and immune cells in the TME of ovarian cancer. TIMER (https://cistrome.shinyapps.io/timer/) database includes 32 types of cancers from The Cancer Genome Atlas (TCGA) with 10897 samples from online resources [75]. The TISIDB (http://cis.hku.hk/TISIDB/index.php) is a database that integrates multiple heterogeneous data types for providing information about the tumor and immune system interaction [76]. The GEPIA database (http://gepia.cancer-pku.cn/index.html) contains information on cancer and normal gene expression profiling, data from TCGA, and The Genotype-Tissue Expression (GTEx) databases [77]. Tumor-infiltrating immune cells from GSE51373 and GSE103737 cohorts were calculated using the CIBERSORT algorithm, which allows researchers to investigate the 22 immune cell phenotypes in complicated tissues based on the standardized gene expression data [78]. CIBERSORT was run following these parameters: LM22 signature gene file, and 1000 permutations. We further compared the proportions of immune cells in TIPE2 high- and low-expressing ovarian cancer samples.

### Statistical analysis

Each experiment was carried out at least three times. GraphPad Prism, version 9.0 software (GraphPad, Inc., USA) was used for statistical analysis. The images were analyzed via ImageJ (National Institutes of Health, Bethesda, USA) software. Normally distributed data were presented as mean ± standard deviation (SD). Statistical significance between the two groups was determined by Student’s t-test. TIPE2 mRNA expression was calculated using the following formula: 2-ΔCt [ΔCt=Ct(target)-Ct(β-actin)]. p < 0.05 was judged statistically significant for all data with the following notations: *p < 0.05, **p < 0.01, ***p < 0.001, ****p< 0.0001.
